# Parental exposure to ocean acidification impacts gamete production and physiology but not offspring performance in *Nematostella vectensis*

**DOI:** 10.1242/bio.059746

**Published:** 2023-02-28

**Authors:** Benjamin H. Glass, Angela H. Schmitt, Kristen T. Brown, Kelsey F. Speer, Katie L. Barott

**Affiliations:** Department of Biology, University of Pennsylvania, Philadelphia, PA 19104, USA

**Keywords:** Climate change, Acclimatization, Carryover effects, Reproduction, Development, Cnidarian

## Abstract

Ocean acidification (OA) resulting from anthropogenic CO_2_ emissions is impairing the reproduction of marine organisms. While parental exposure to OA can protect offspring via carryover effects, this phenomenon is poorly understood in many marine invertebrate taxa. Here, we examined how parental exposure to acidified (pH 7.40) versus ambient (pH 7.72) seawater influenced reproduction and offspring performance across six gametogenic cycles (13 weeks) in the estuarine sea anemone *Nematostella vectensis*. Females exhibited reproductive plasticity under acidic conditions, releasing significantly fewer but larger eggs compared to ambient females after 4 weeks of exposure, and larger eggs in two of the four following spawning cycles despite recovering fecundity, indicating long-term acclimatization and greater investment in eggs. Males showed no changes in fecundity under acidic conditions but produced a greater percentage of sperm with high mitochondrial membrane potential (MMP; a proxy for elevated motility), which corresponded with higher fertilization rates relative to ambient males. Finally, parental exposure to acidic conditions did not significantly influence offspring development rates, respiration rates, or heat tolerance. Overall, this study demonstrates that parental exposure to acidic conditions impacts gamete production and physiology but not offspring performance in *N*. *vectensis*, suggesting that increased investment in individual gametes may promote fitness.

## Teaser

**Summary:** Ocean acidification (OA) threatens the reproduction of marine invertebrates. We investigated how simulated OA affected the reproduction of a model sea anemone, revealing effects with mixed implications for fitness.

## INTRODUCTION

Global anthropogenic carbon dioxide (CO_2_) emissions are significantly altering the carbonate chemistry of the ocean, which has absorbed more than 30% of anthropogenic CO_2_ (Doney et al., 2009). As a result, the average global ocean surface pH has dropped by 0.1 units since the 19th century in a process termed ocean acidification (OA), and is expected to continue decreasing by as much as 0.3–0.5 units by 2100 (Doney et al., 2009; Godbold and Calosi, 2013). OA has already had negative impacts on a diversity of marine organisms, which are facing increased energy costs of maintaining key processes such as growth and reproduction in the face of external pH stress (Hoegh-Guldberg et al., 2007; Kroeker et al., 2013; Vargas et al., 2022). Understanding the effects of OA on marine invertebrate reproduction in particular is essential for predicting organismal fitness in future seas, yet these effects differ both within and between species (Foo and Byrne, 2017; Hill and Hoogenboom, 2022; Kroeker et al., 2010; Padilla-Gamiño et al., 2022), emphasizing a need to examine responses in a broader diversity of life stages and taxa. In particular, relatively little is known about how OA affects the reproduction of non-calcifying species in the ecologically important phylum Cnidaria, such as sea anemones (but see Albright, 2011; Hill and Hoogenboom, 2022; Padilla-Gamiño et al., 2022). Many cnidarians release their gametes directly into the water column via broadcast spawning, making them particularly vulnerable to OA (Byrne et al., 2020; Foo and Byrne, 2017; Przeslawski et al., 2015). However, parental exposure to environmental stressors like OA can promote offspring resilience via intergenerational acclimatization, a product of parental carryover (i.e. legacy) effects (Jensen et al., 2014; Putnam, 2021). The extent to which direct effects on reproductive performance and parental carryover effects on gamete and offspring performance can promote the resilience of cnidarians to OA remains an outstanding question yet is critical for understanding the fates of these species in a changing ocean.

The direct impacts of OA on cnidarian reproduction vary within and between species and depend on the duration of exposure. For example, exposure to acidic conditions reduces both female fecundity and egg size in the soft coral *Primnoa pacifica* (Rossin et al., 2019), while these metrics are unaffected in females of the corals *Leptopsammia pruvoti* (Gizzi et al., 2017) and *Balanophyllia europaea* (Caroselli et al., 2019). Additionally, low pH does not affect male or female fecundity, but does delay spermary development in the coral *Astroides calycularis* (Marchini et al., 2021). This heterogeneity in cnidarian responses to acidic conditions mirrors results from other phyla. For example, in a diversity of marine annelids, molluscs, and crustaceans, exposure to OA has species- and sex-specific effects on male and female fecundity and egg sizes that can be positive, negative, or neutral for fitness (Foo and Byrne, 2017; Kroeker et al., 2010, 2013; Leung et al., 2022; Vargas et al., 2022), highlighting the variable nature of OA effects across these diverse taxa.

As with direct effects of OA on cnidarian reproduction, parental carryover effects of OA exposure also vary between species. For example, after parental exposure to acidic conditions, larvae of the brooding coral *Pocillopora damicornis* exhibit metabolic enhancement as well as increased survivorship and settlement under low pH conditions relative to control larvae, indicating beneficial carryover effects on offspring performance following parental exposure (Putnam and Gates, 2015; Putnam et al., 2020). Additionally, larvae of the coral *Stylophora pistillata* are resistant to low pH following parental exposure to simulated ocean warming and OA (Bellworthy et al., 2019). Positive parental carryover effects have also been observed in molluscs and echinoderms, which exhibit increases in larval growth, development, and biomineralization, perhaps due to increases in egg provisioning following parental OA exposure (Maboloc and Chan, 2021; Marčeta et al., 2022; McNally et al., 2022; Thibault et al., 2020; Zhao et al., 2020), suggesting that this may be an evolutionarily conserved mechanism of resilience. However, parental OA exposure leads to decreases in fecundity in the slipper limpet *Crepidula onyx* (Maboloc and Chan, 2021), as well as decreases in larval survival in the sea urchin *Paracentrotus lividus* (Marčeta et al., 2022), demonstrating that parental carryover effects can also be detrimental for offspring and are thus difficult to predict or generalize across taxa.

Understanding the direction and magnitude of parental carryover effects of OA on cnidarian gametes and larvae is particularly important given that acidic conditions are also predicted to have direct impacts on these important life stages. For example, acidified seawater dramatically reduces sperm motility in a diversity of marine invertebrates including echinoderms, molluscs, ascidians, and cnidarians (Esposito et al., 2020; Hudson and Sewell, 2022; Marčeta et al., 2022; Morita et al., 2010). While parental carryover effects manifested through changes in gamete physiology might ameliorate the harmful effects of OA on early life stages, the role of these effects in isolation from offspring phenotypic plasticity has rarely been investigated in cnidarians (Albright, 2011; Padilla-Gamiño et al., 2022), largely due to the difficulty of breeding these species in the laboratory. The sea anemone *Nematostella vectensis* has emerged as an important model species for investigating the reproduction and early development of early-diverging marine invertebrates (Darling et al., 2005; Layden et al., 2016), and may be a useful species for addressing this question. Native to the United States Atlantic coast (Stefanik et al., 2013), *N. vectensis* is a dioecious, broadcast spawning cnidarian, and is typically found in salt marsh pools characterized by large fluctuations in salinity, temperature and pH that vary on both daily and seasonal scales (Poach et al., 2019; Reitzel et al., 2013; Rosenau et al., 2021). This natural history makes *N. vectensis* a potentially informative model for studying reproductive and developmental plasticity in a changing climate. Indeed, *N. vectensis* anemones reared at elevated temperatures produce larvae with increased thermal tolerance (Rivera et al., 2021), indicating that parental carryover effects may play an important role in acclimatization to stressors in this species.

Here, we explored the impacts of OA on the reproduction of *N. vectensis* and the potential role of carryover effects in influencing performance of early life stages. While the effects of OA on reproduction and early development are variable across species, meta-analyses of responses across phyla predict overall negative effects of OA on survival, growth, reproduction, and other processes (Kroeker et al., 2010), and suggest that cnidarians like *N. vectensis* are among the most threatened groups under OA (Padilla-Gamiño et al., 2022). Given this information, we hypothesized that parental exposure to acidification stress would negatively impact gamete physiology and larval performance in *N. vectensis* due to trade-offs between investment in reproduction and other processes (e.g. growth) under OA conditions. To test this hypothesis, adult male and female anemones were exposed to ambient (pH 7.72) and acidified (pH 7.40) seawater conditions over six gametogenic cycles (13 weeks), representing the high and low end of pH values transiently experienced by *N. vectensis* in its natural habitat (Baumann et al., 2015; Velinsky et al., 2017). Highly variable environments such as the estuaries inhabited by *N. vectensis* are expected to experience low pH extremes more frequently due to OA than is observed in the present day (Gaitán-Espitia et al., 2017; Vargas et al., 2022), and thus sustained exposure to pH 7.40 represents future OA conditions likely to be increasingly experienced by these animals. Spawning activity as well as gamete and offspring performance were assessed at each spawning event. For all gamete and offspring metrics, performance was assayed under ambient conditions, allowing us to isolate parental carryover effects from developmental effects and offspring phenotypic plasticity. This study provides an important step toward understanding cnidarian reproduction and development in future seas.

## RESULTS

Details of all statistical tests performed with corresponding significance information are summarized in Table S1. All post-hoc pairwise comparisons were performed via Tukey's Honest Significant Difference (HSD) test, and all values reported are means±s.e.

### Experimental treatment conditions

**Fig. 1.**
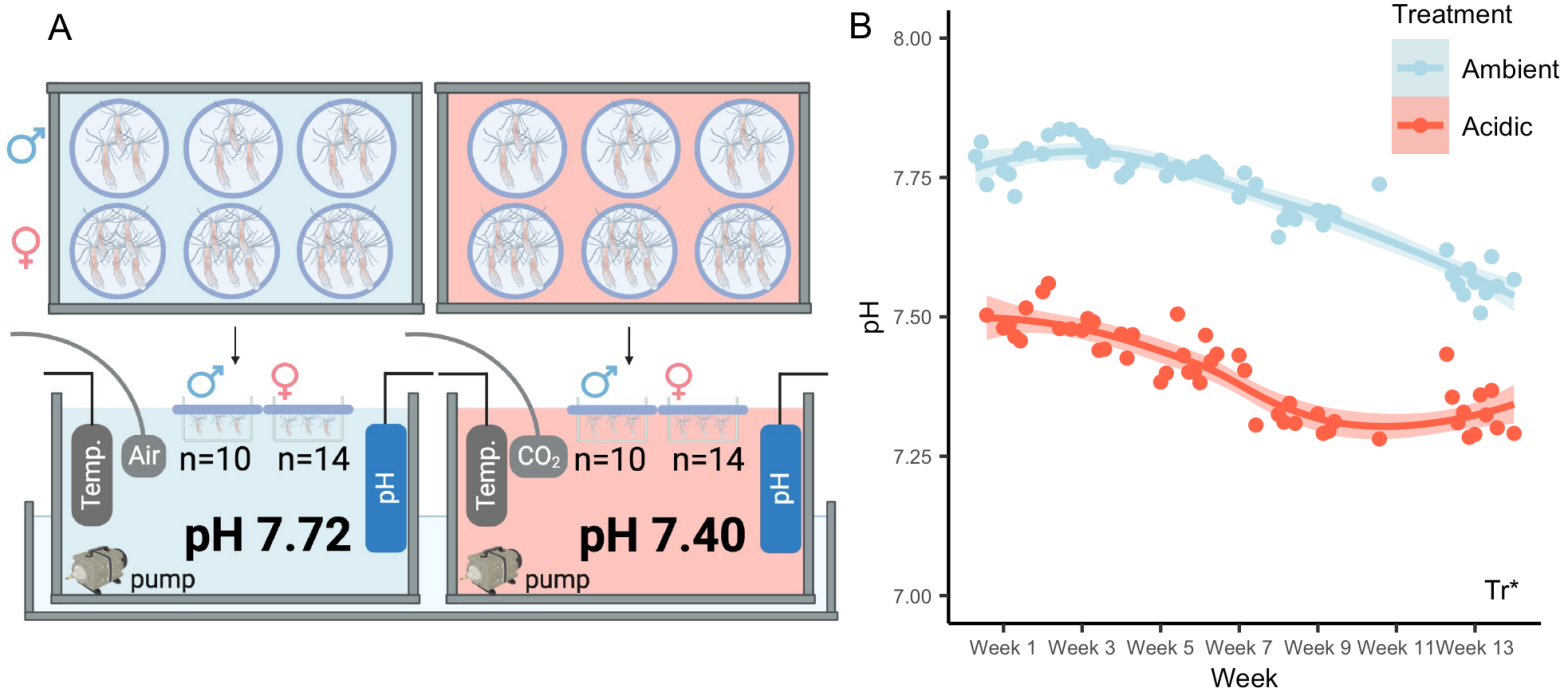
**Experimental setup.** (A) Two tubs were used to generate the experimental conditions. Each tub was equipped with a temperature probe, pH probe, and water pump. For the acidic treatment (red), CO_2_ was added via a solenoid valve and CO_2_ tank under control of an Apex monitoring system, while room air was bubbled in continuously for the ambient treatment (blue). The tubs were placed within a recirculating freshwater bath (bottom) maintained at 18°C using an aquarium chiller. Anemones (*N*=10 males and 14 females per treatment) were separated by sex and placed into three replicate plastic containers per sex with 100 μm mesh bottoms kept afloat in the tubs by a foam ring (lilac). (B) Seawater pH data for the ambient (blue) and acidic (red) treatments over the course of the experiment. Points represent average pH values for duplicate water samples collected approximately every three days throughout the experiment. Lines depict loess curves with shaded regions representing standard errors of curve fits; asterisk indicates treatment (Tr) as a significant model term (*P*<0.05; Type II ANOVA).

The mean pH of the acidic tub was significantly lower (7.40±0.01) than that of the ambient tub (7.72±0.01; *P*<0.001; Fig. 1A,B) throughout the duration of the experiment. While there was a decline in the pH of both tubs over the course of the experiment, the ambient treatment conditions were consistently higher than the acidic treatment throughout the experiment (Fig. 1B). In addition, the mean *p*CO_2_ was significantly higher in the acidic tub (1375.2±135.9 μatm) compared to the ambient tub (494.4±33.2 μatm; *P*<0.001). All seawater carbonate chemistry parameters are summarized in Table 1.

**Table 1.**
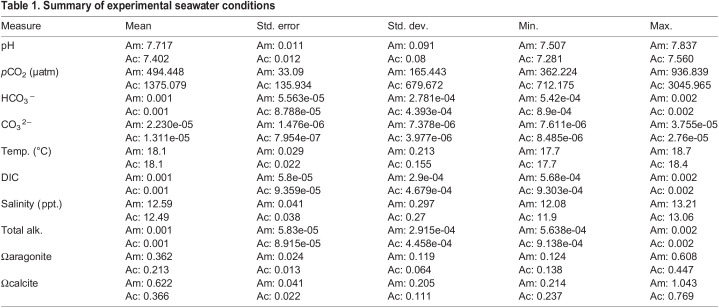
Summary of experimental seawater conditions

### Female fecundity and egg sizes

**Fig. 2.**
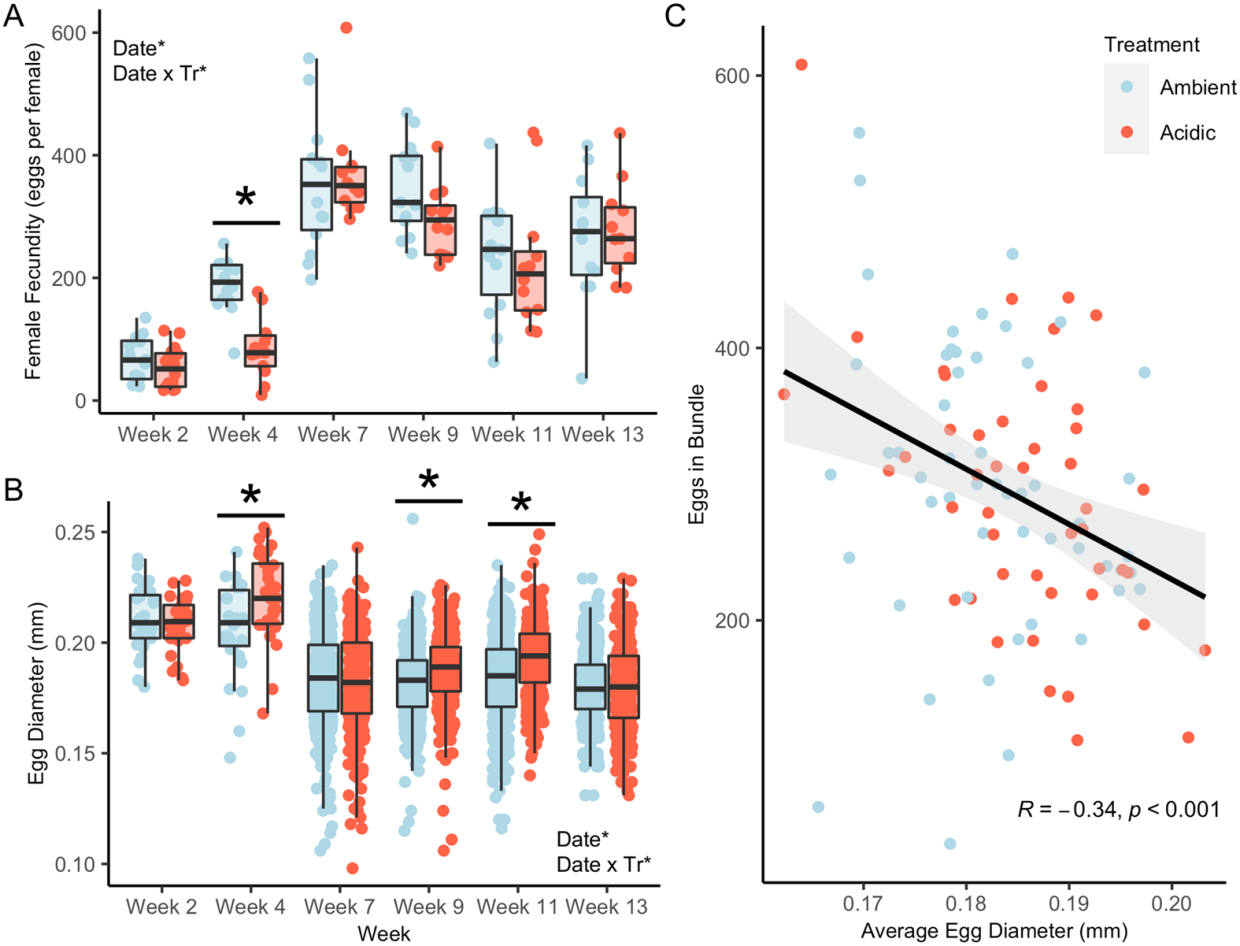
**Exposure to seawater acidification impacted female reproduction.** (A) Female fecundity of *N. vectensis* over the course of 13 weeks of exposure to acidic (pH 7.40) versus ambient (pH 7.72) conditions. Asterisks indicate date and the interaction between date and treatment (Date×Tr) as significant (*P*<0.05; Type III ANOVA) model terms; over bar, asterisk also indicates significant pairwise difference in means between treatments (*P*<0.05; Tukey's HSD). (B) Egg diameters (*N*=34–681 eggs). Asterisks again indicate significant (*P*<0.05) model terms and pairwise differences in means between treatments. (C) Relationship between egg size and number of eggs per bundle (*N*=99 bundles). Line and shaded region depict a linear model (number∼size) with standard error.

At the first spawning in week 2, ambient females (*N*=14) produced a mean of 68±20 eggs while acidic females (*N*=14) produced 54±19 eggs, and there was no significant difference between treatments (*P*=0.599; Fig. 2A). At the second spawning in week 4, egg production significantly increased for both groups compared to week 2 (*P*<0.001 for both), but females from the acidic treatment produced significantly fewer eggs (85±21) than their ambient counterparts (188±20; *P*<0.001; Fig. 2A). Egg production increased again at week 7 compared to week 4 for both groups (*P*<0.001 for both), then largely leveled off for the remainder of the experiment (Fig. 2A). Egg diameter showed no significant difference between treatments at the first spawning in week 2 (*P*=0.619). However, at the next spawning cycle in week 4, females from the acidic treatment produced significantly larger diameter eggs (0.221±0.003 mm) than their ambient counterparts (0.206±0.003 mm; *P*=0.002). At the next spawning in week 7, there was no significant difference in egg size between the two treatments, but both groups produced significantly smaller eggs compared to weeks 2 and 4 (*P*<0.001 for both). In weeks 9 and 11, acidic females again produced significantly larger eggs than ambient females: 0.187±0.001 mm for acidic versus 0.181±0.001 mm for ambient in week 9 (*P*<0.001), and 0.193±0.001 mm for acidic versus 0.183±0.001 mm for ambient in week 11 (*P*<0.001; Fig. 2B). Finally, the number of eggs per bundle showed a significant negative relationship with egg size when pooled across the experiment (R=−0.34; *P*<0.001; Type II ANOVA; Fig. 2C), and treatment did not significantly affect this relationship (*P*=0.77).

### Male fecundity and sperm performance

**Fig. 3.**
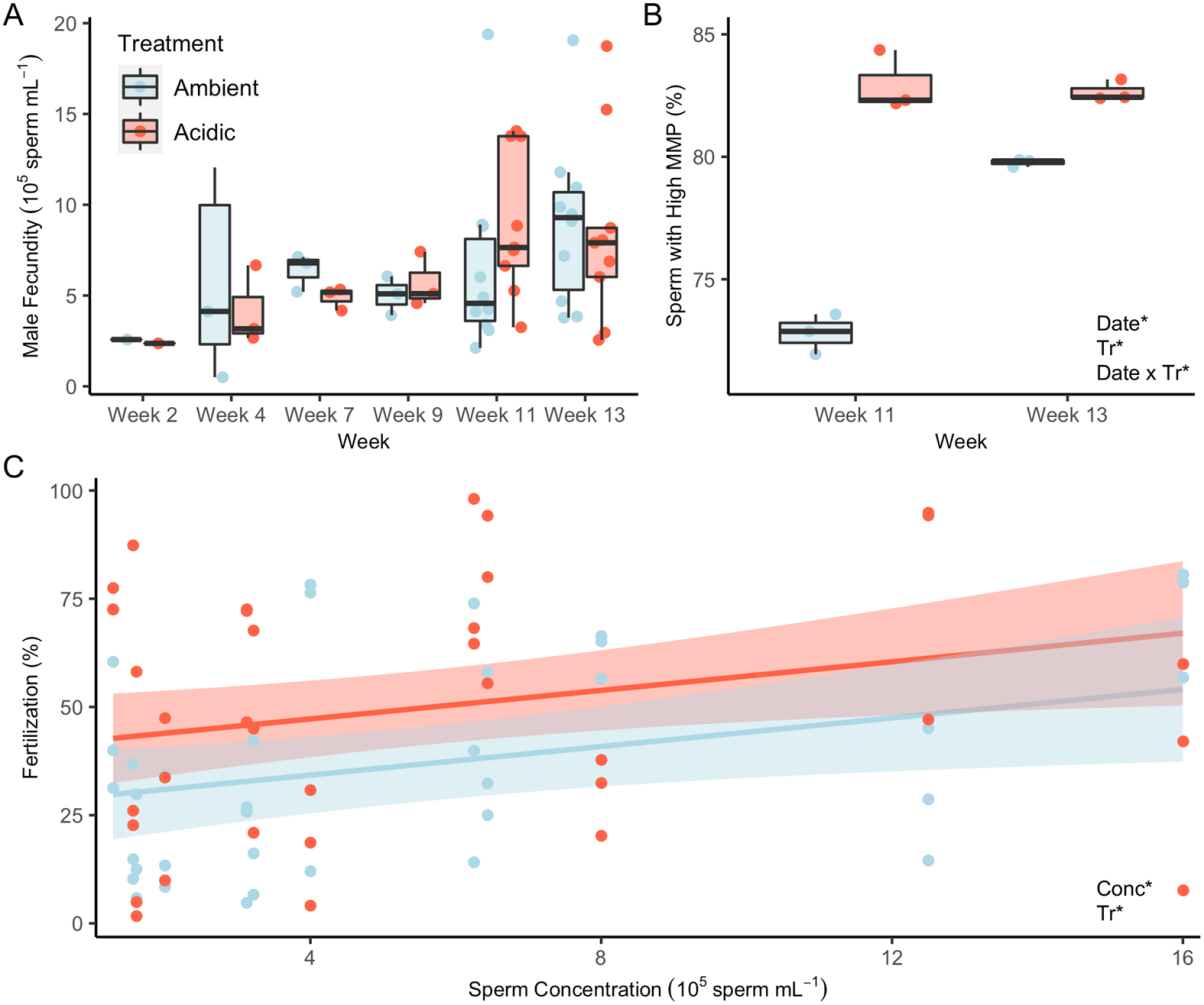
**Exposure to acidification enhanced male reproductive performance.** (A) Male fecundity for each spawning date (*N*=1–3 groups for weeks 2–9 or 9–10 males for weeks 11–13). (B) Percentage of sperm (*N*=3 replicates of pooled sperm per spawning cycle) displaying high MMP. Asterisks indicate significant (*P*<0.05; Type III ANOVA) model terms (Tr=treatment) and, when over bars, significant pairwise differences (*P*<0.05; Tukey's HSD). (C) Linear relationship between sperm concentration and fertilization percentage (*N*=72 assays). Asterisks again indicate significant model terms (*P*<0.05; Tukey's HSD). Lines depict regressions for data predicted with linear model; shading represents a 95% confidence interval for predictions.

Male fecundity increased over the 13-week treatment period, though this was not statistically significant (*P*=0.182; Type II ANOVA), likely due to small sample sizes (*N*=1–3 groups of males) for the initial spawning events (Fig. 3A). Across all dates, ambient males (*N*=10) produced a mean of 5.75±0.947×10^5^ sperm per male while acidic males (*N*=10) produced a mean of 6.06±0.959×10^5^ sperm per male, and no significant differences were detected between treatments (*P*=0.789; Type II ANOVA). Males from the acidic treatment released 83±0.45% and 82.7±0.45% sperm with high mitochondrial membrane potential (MMP; a proxy for elevated motility) in weeks 11 and 13, respectively. For both of these spawning events, percentages of sperm with high MMP were significantly higher for males from the acidic group compared to the ambient group (*P*<0.05 for both weeks), with ambient males releasing 72.8±0.45% and 79.8±0.45% sperm with high MMP, respectively (Fig. 3B). For ambient males, time was a significant factor, as these males produced higher percentages of sperm with high MMP in week 13 as compared to week 11 (*P*<0.001; Fig. 3B). Finally, fertilization rates were significantly higher for acidic parents relative to ambient parents across a range of sperm concentrations (*P*=0.037; Type II ANOVA; Fig. 3C), and fertilization rates increased significantly with increasing sperm concentration in both treatment groups (*P*=0.018; Type II ANOVA).

### Larval performance

**Fig. 4.**
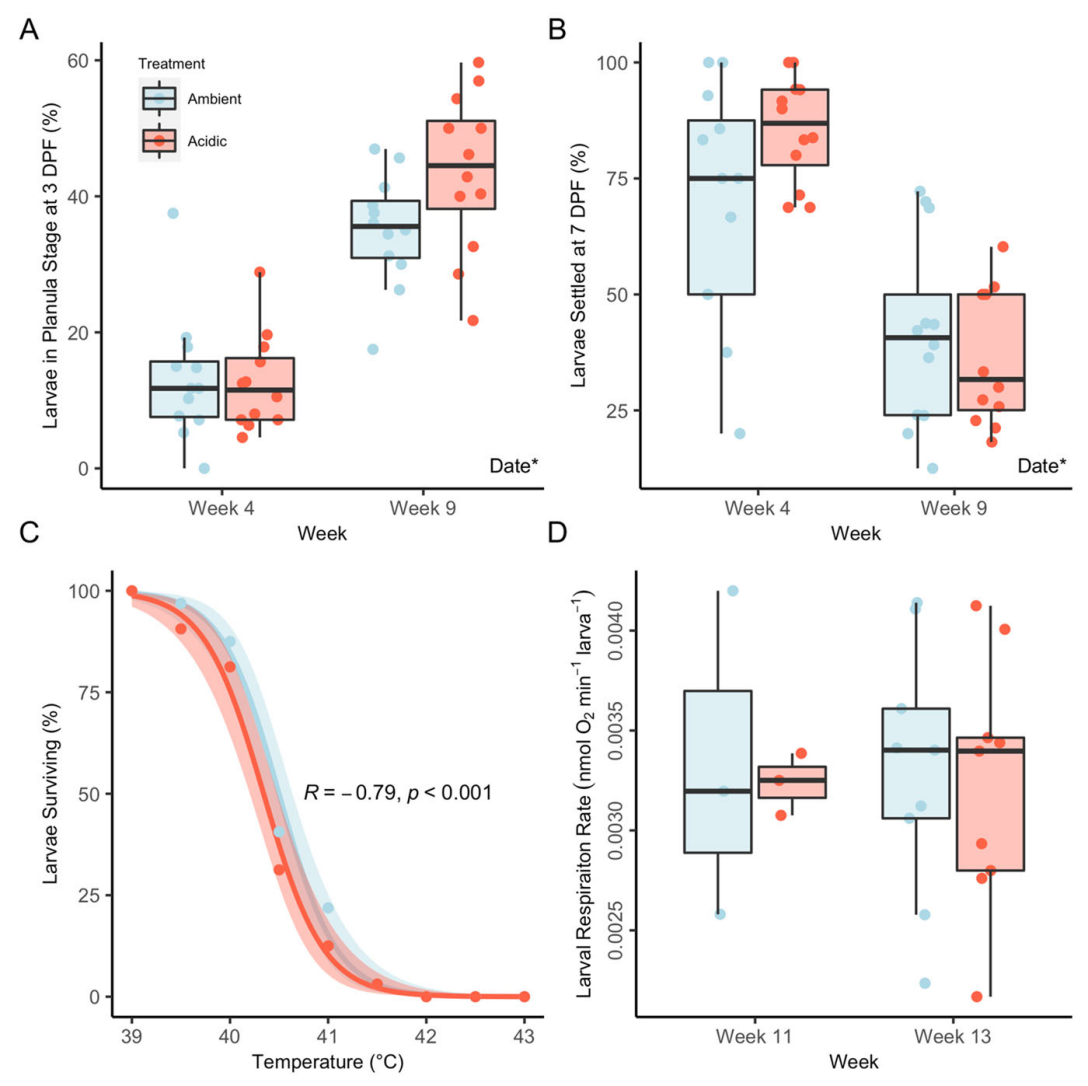
**Parental exposure to seawater acidification did not significantly impact larval performance under ambient conditions.** (A) Percentage of larvae (*N*=24 cohorts) reaching planula stage by 3 DPF. Asterisk indicates date as a significant (*P*<0.05; Type II ANOVA) model term. (B) Percentage of larvae (*N*=24 cohorts) reaching settlement by 7 DPF. Asterisk indicates date as a significant (*P*<0.05; Type II ANOVA) model term. (C) Survival rates for 3 DPF larvae (*N*=576) exposed to a short heat stress. Lines depict generalized linear models, and shading represents the standard error. Inset metrics apply to both curves, with R indicating the correlation coefficient and *P* indicating significance of temperature (Type II ANOVA). (D) Larval respiration rates (*N*=60–270) at 3 DPF.

Larval developmental progression was unaffected by parental treatment at either the planula stage [3 days post fertilization (DPF); *P*=0.152; Type II ANOVA; Fig. 4A] or the settlement stage (7 DPF; *P*=0.319; Fig. 4B) for both raw and arcsine-square root transformed data, of which the former were used for visualization. Spawning date, however, was a significant factor for developmental timing, although in opposite directions between the two stages. Specifically, a higher percentage of larvae reached the planula stage by 3 DPF at the week 9 spawning (39.3±1.92%) compared to week 4 (12.9±1.92%; *P*<0.001; Fig. 4A), whereas a higher percentage of larvae reached the settlement stage at 7 DPF in week 4 (77.6±3.93%) compared to week 9 (39±3.93%; *P*<0.001; Fig. 4B). Larval heat tolerance, assessed at 3 DPF under ambient conditions, was not affected by parental treatment (*P*=0.106; Type II ANOVA), and the observed LT50 s were approximately 40.5±0.06°C for ambient and 40.34±0.06°C for acidic larvae (Fig. 4C). Similarly, larval respiration rates assessed at 3 DPF under ambient conditions did not differ between treatments (*P*=0.781; Type II ANOVA) or between weeks (*P*=0.954; Fig. 4D). It is important to note that larval respiration data were not normalized to larval size; however, larvae were not noticeably different in size between the two parental treatments.

### Adult respiration

**Fig. 5.**
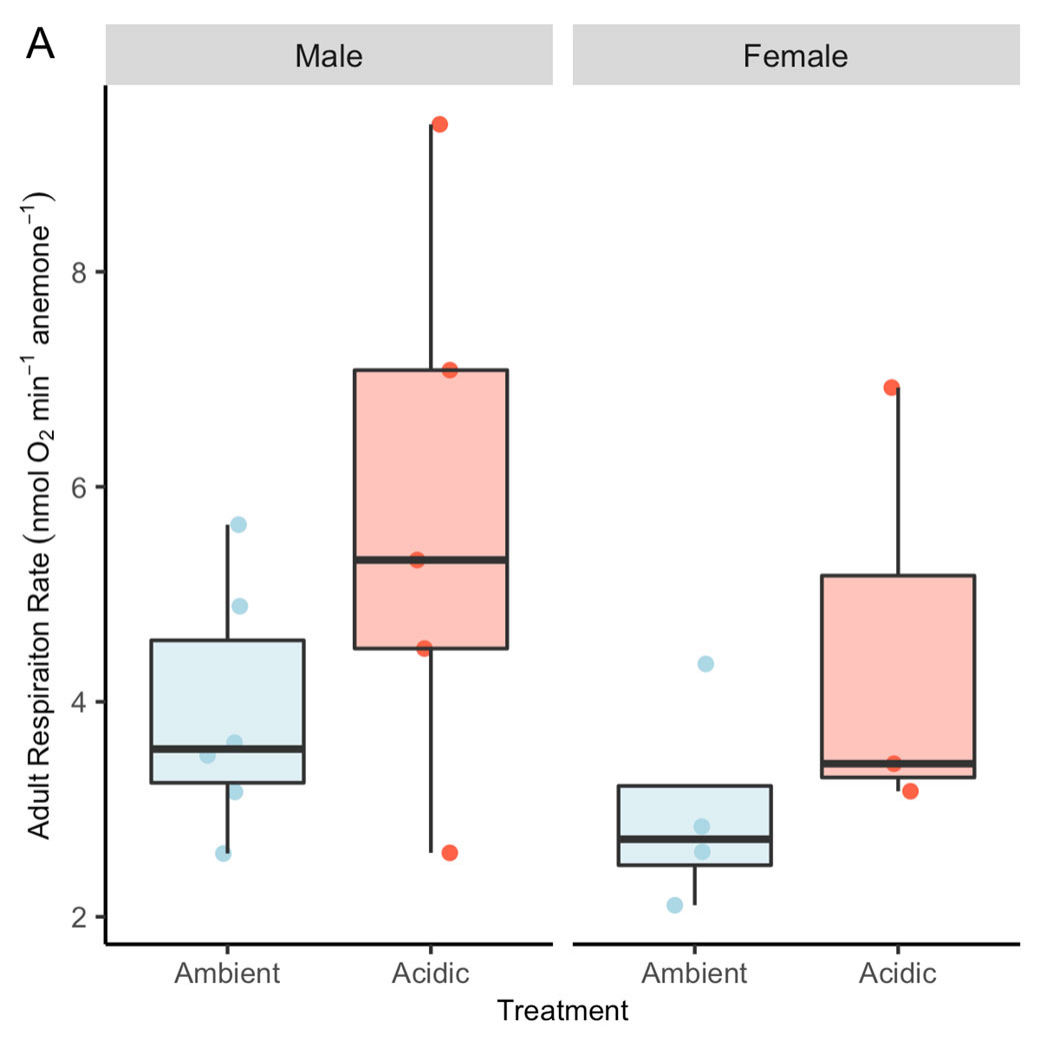
**Exposure to decreased seawater pH did not significantly affect adult respiration.** (A) Respiration rates of adult male and female anemones after ∼14 weeks of exposure to ambient (pH 7.40) versus acidic (pH 7.72) experimental conditions. Neither treatment (*P*=0.051; Type II ANOVA) nor sex (*P*=0.218) was significant.

Adult anemone respiration rates in the respective treatment conditions were not significantly affected by either sex or treatment after 14 weeks of exposure (*P*>0.05; Type II ANOVA; Fig. 5A), though anemones from the acidic treatment displayed a trend of increased respiration rates compared to ambient counterparts that was marginally significant (*P*=0.051). While adults in both treatments were of similar size, biomass was not quantified, and thus respiration rates were not normalized to anemone size.

## DISCUSSION

OA is predicted to have harmful effects on the reproduction and early development of marine invertebrates, especially for species with gametes and offspring that are directly exposed to seawater conditions during broadcast spawning (Byrne et al., 2020; Foo and Byrne, 2017; Kroeker et al., 2010; Padilla-Gamiño et al., 2022). Here, we found that long-term exposure (13 weeks; six gametogenic cycles) to low pH (pH 7.40, simulating an increase in the frequency of low pH events under OA) had a number of effects on the reproduction of both sexes of the sea anemone *N. vectensis*. Our results show adult acclimatization and shifts in reproductive energy allocation that may promote offspring performance. Specifically, females exposed to acidic conditions released significantly larger eggs across several spawning cycles, while males exposed to acidic conditions produced a greater percentage of sperm with high MMP (a proxy for increased motility). These differences in gamete physiology corresponded with elevated fertilization success for acidic parents relative to ambient parents. Finally, parental exposure to acidic conditions did not appear to significantly influence larval development, respiration, or heat tolerance, indicating an absence of harmful carryover effects for offspring performance. Taken together, these results provide some of the first evidence for parental carryover effects induced by simulated OA conditions in a non-calcifying cnidarian, improving our understanding of how future OA may affect this ecologically critical group of species.

### Acidic conditions led to increased maternal reproductive investment

Females exposed to acidic conditions produced significantly fewer but larger eggs than those under ambient conditions after 4 weeks of exposure, indicating an initial shift in maternal investment under pH stress. Decreases in female fecundity under acidic conditions have also been observed in the slipper limpet *Crepidula onyx* (Maboloc and Chan, 2021) and the annelid *Ophryotrocha robusta* (Thibault et al., 2020), though this response is absent in other annelids (Chakravarti et al., 2016) and stony corals (Padilla-Gamiño et al., 2022), emphasizing the species-specific nature of OA effects on female fecundity. It is also worth noting the effect of time on female fecundity, as females in both ambient and acidic conditions displayed increases in egg production over the first 7 weeks of the experiment, likely due to initial growth resulting from increased access to food in the experimental treatment conditions. Fecundity remained stable and not significantly different for females in the ambient and acidic treatments for the remainder of the experiment, even as pH levels in both treatments decreased slightly during this time frame, suggesting that females in both treatments were able to acclimatize to increasing pH stress.

Interestingly, females under acidic conditions produced significantly larger eggs than females under ambient conditions in half of all spawning events, despite a lack of differences in egg production following prolonged exposure to acidified conditions. This indicates further acclimatization and a shift in maternal energy investment toward larger eggs even after fecundity had recovered. Investment in larger eggs under acidic conditions could be an adaptation evolved by *N. vectensis* in response to low pH stress transiently but frequently experienced in its estuarine habitat (Baumann et al., 2015; Velinsky et al., 2017). Larger eggs are often correlated with a higher probability of fertilization (Allen and Marshall, 2014; Marshall and Keough, 2007; Podolsky and Strathmann, 1996), and could thus promote fertilization in the face of decreased sperm motility, which can be caused by acidic conditions (Esposito et al., 2020; Hudson and Sewell, 2022; Leuchtenberger et al., 2022; Morita et al., 2010; Nakamura and Morita, 2012). Increased maternal investment in larger eggs could also ameliorate any increase in offspring energy demands required for maintaining homeostasis under acidified conditions, as seen in other species (Maboloc and Chan, 2021; Nakamura et al., 2011; Przeslawski et al., 2015; Yuan et al., 2018). Since egg size is a proxy for energy reserves in lecithotrophic marine invertebrates including *N. vectensis* (Holcomb et al., 2012; Levitan et al., 2015; Marshall and Keough, 2007; Moran and McAlister, 2009), larger eggs are likely to contain greater energetic resources in this species. Increases in egg sizes without changes in fecundity under acidic conditions have also been observed in other marine invertebrates, including the annelid *Ophryotrocha labronica* (Chakravarti et al., 2016) and sea urchin *Sterechinus neumayeri* (Suckling et al., 2015). Interestingly, *S. neumayeri* displays increases in egg sizes without changes in fecundity after long- but not short-term exposure to acidic conditions (Suckling et al., 2015), mirroring what we observed here in *N. vectensis*. Thus, our results reveal a potentially conserved response to long-term exposure to acidic conditions.

Tracking the numbers and sizes of eggs produced by all females over the course of the experiment revealed a trade-off between individual egg production and egg size, which has been suggested to occur for *N. vectensis* (Rivera et al., 2021) but, to our knowledge, had not been empirically confirmed. Across a broad diversity of animal species, increases in egg production are often associated with decreases in egg size (Closs et al., 2013; Fleming and Gross, 1990; Hazraty-Kari et al., 2022; Hein et al., 2018; Jonsson and Jonsson, 1999; Pellerin et al., 2016; Podolsky and Strathmann, 1996; Rowe, 1994), which has implications for offspring development and survival (Allen and Marshall, 2014; Closs et al., 2013; Hazraty-Kari et al., 2022; Marshall and Keough, 2007). Indeed, we found that larval settlement rates were higher when mean egg sizes were larger, though we cannot strictly establish egg size as a causal factor. Furthermore, while we did start the experiment with anemones that were approximately the same size across both treatment groups, interpretation of results related to fecundity is complicated by the fact that neither size nor growth rates were quantified throughout the experiment. Nonetheless, these results suggest a complex acclimatory response to OA meriting further investigation, and highlight the need for long-term studies encompassing multiple gametogenic cycles, which may more accurately uncover conserved organismal responses to stress compared to short-term experiments.

### Males exposed to seawater acidification produced sperm with improved performance

Compared to energy rich eggs, sperm are much less costly to produce, so male fecundity is predicted to be less sensitive to environmental stress (Levitan, 1993). In accordance with this hypothesis, we observed no differences in sperm production between males exposed to ambient or acidic conditions. These results are similar to patterns observed in other cnidarians, including *Leptopsammia pruvoti* and *Primnoa pacifica*, which also show no significant impact of OA conditions on male fecundity (Gizzi et al., 2017; Rossin et al., 2019). In contrast, males of the sea urchin *Echinometra mathaei* do show decreased spawning ability under low pH (Uthicke et al., 2013), suggesting that effects of OA conditions on male fecundity differ between phyla.

Interestingly, male anemones exposed to acidic conditions produced a larger percentage of sperm displaying high MMP compared to males under ambient conditions, indicating metabolic enhancement that could improve sperm performance and thus fitness. Additionally, the MMP of sperm produced by males under ambient conditions increased between weeks 11 and 13, corresponding with a slight decline in pH in the ambient treatment, further supporting the hypothesis that the observed increases in MMP resulted from male exposure to acidic conditions. For marine broadcast spawners, energy used for sperm motility is generated as adenosine triphosphate (ATP) by sperm mitochondria, so a sperm cell's overall MMP is a proxy for sperm motility and is a key indicator of sperm mitochondrial quality (Agnihotri et al., 2016). Indeed, high sperm MMP has been shown to be tightly correlated with increased sperm motility in at least two other cnidarian species (Henley et al., 2021). Sperm motility often correlates with fertilization success, and although we did not measure sperm MMP and fertilization in the same spawning event, we did observe increased fertilization rates in gametes from parents in the acidic treatment. These data suggest that male investment in sperm quality promotes fertilization success and fitness under acidic conditions for *N. vectensis*. However, direct exposure of sperm to acidic conditions can decrease motility (Esposito et al., 2020; Hudson and Sewell, 2022; Morita et al., 2010), MMP (Esposito et al., 2020), and fertilization (Havenhand et al., 2008), possibly due to the role of cytosolic alkalinization in the activation of sperm motility (Nishigaki et al., 2014; Speer et al., 2021). This could mean that the positive paternal carryover effect of elevated sperm MMP and fertilization success might be negated if sperm are also exposed to low pH. However, sperm from *P. lividus* males raised under acidic conditions showed increased longevity even in acidified seawater (Marčeta et al., 2022), suggesting that positive parental carryover effects on sperm performance (e.g. increased MMP, as observed here) might be robust even when sperm are exposed to acidic conditions upon spawning. Given that *N. vectensis* transiently experiences acidic conditions in its estuarine habitats (Baumann et al., 2015; Velinsky et al., 2017), increases in sperm MMP could be an example of evolved adaptive plasticity. However, further research is needed to investigate if the carryover effect of elevated MMP is a mechanism of resilience following gamete exposure to pH stress in order to clarify how future OA conditions might impact reproduction through combined parental and direct effects on sperm physiology. Finally, further investigation is also needed to clarify the role of male genotype in driving stress responses and parental carryover effects, as marine invertebrates can display individual heterogeneity in sperm performance following exposure to pH stress (Lymbery et al., 2022).

### Larval performance was unaffected by parental exposure to acidic conditions

In the face of intensifying global change, parental exposure to ocean warming and acidification may promote offspring resilience through transgenerational plasticity mediated via parental carryover effects (Bellworthy et al., 2019; Hazraty-Kari et al., 2022; Maboloc and Chan, 2021; Marčeta et al., 2022; Minuti et al., 2022; Putnam et al., 2020). We found that parental exposure to OA had no impact on the rate of progression to the planula or settlement stage of larval development in *N. vectensis*, indicating a lack of carryover effects of low pH on the timing of development. In addition, larval respiration was not affected by low pH, indicating a possible absence of carryover effects on this phenotype, though these data were not normalized to larval size and should be interpreted with caution. Larval respiration rates can have mixed implications for survival, since increased cellular respiration produces more energy to deal with stressful conditions but also results in faster consumption of energy reserves (Cumbo et al., 2013). When exposed to OA, larvae of the coral *Pocillopora damicornis* show increased metabolism, settlement, and survivorship under OA conditions (Putnam and Gates, 2015; Putnam et al., 2020). However, *P. damicornis* larvae are brooded and therefore not exposed to seawater until they are fully developed, meaning that parental carryover effects operate concurrently with larval phenotypic plasticity. Here, we investigated larval phenotypes under ambient conditions in an externally fertilizing and developing species, allowing us to specifically isolate parental carryover effects from offspring phenotypic plasticity. Our results indicate some metabolic resilience for the first motile life stage in *N. vectensis* following long-term parental exposure to acidic conditions, which is encouraging for this species' future persistence.

We also found that larval heat tolerance was unaffected by treatment, further supporting possible larval resilience following parental OA exposure. Originally, we hypothesized that parental exposure to OA stress might negatively impact offspring performance via parental carryover effects (Marčeta et al., 2022; Putnam, 2021), for example if stress interfered with gametogenesis. We used heat tolerance as one measure of offspring quality because this phenotype is indicative of the ability of larvae to tolerate abiotic stress (Rivera et al., 2021), which might be diminished if parental stress exposure negatively affected gametogenesis. Heat tolerance is also relevant in an ecological context, as ocean warming is occurring in concert with OA (Kroeker et al., 2013). While our data suggest that parental exposure to OA is unlikely to sensitize *N. vectensis* larvae to ocean warming, carryover effects of exposure to the combination of elevated temperatures and acidification have not been investigated, and future research could investigate the influence of these dual stressors in combination to more accurately predict how future climate change might impact larval physiology in this species.

### Parental exposure to acidic conditions has mixed implications for fitness

Overall, the results of this study demonstrate that environmental factors can modulate reproductive physiology and parental carryover effects in *N. vectensis* in ways that have both positive and negative implications for fitness. Both males and females showed reproductive plasticity in response to acidic conditions, but no effects on larval performance were identified. Increased egg sizes combined with higher sperm motility and fertilization success may help *N. vectensis* maintain fitness under increased frequency of low pH events (i.e. future estuary OA conditions), especially considering the lack of observed differences in larval performance. These results lay the groundwork for future research on intra- and intergenerational effects of OA on members of the ecologically and evolutionarily important phylum Cnidaria, which will ultimately help uncover possible mechanisms of resilience for the persistence of these invaluable organisms in the face of continued global change.

## MATERIALS AND METHODS

### Anemone collection and culturing

*Nematostella vectensis* (Stephenson, 1935) anemones were collected from a salt marsh in Brigantine, New Jersey in the fall of 2020. Females were identified by inducing spawning (see below), and 14 individuals that released eggs were chosen as the genotype pool for this experiment. Each female was then horizontally bisected through the body column using a razor blade, resulting in two genotypically identical individuals that were divided between the two experimental groups (ambient and acidic). A clonal male population, also originating from the United States Atlantic coast, was obtained from the laboratory of Dr. Katerina Ragkousi (Amherst College) in the spring of 2021. The male population size was increased via bisection, resulting in a total of 20 genetically identical males for the experiment (*N*=10 per treatment). All anemones were kept in 12 parts per thousand (ppt) artificial seawater (ASW; Instant Ocean Reef Crystals^Ⓡ^ reef salt, Spectrum Brands, Blacksburg, VA, USA) at pH 7.7–8.1 and 18°C. The animals were maintained in a dark incubator (Boekel Scientific, Feasterville-Trevose, PA, USA) and fed approximately every other day with *Artemia nauplii* (Hand and Uhlinger, 1992; Stefanik et al., 2013). The experiment was performed approximately 1–1.5 years after animal collection.

### Experimental conditions

Two 6-L opaque tubs with lids (Rubbermaid, Atlanta, GA, USA) were used to generate the experimental conditions (Fig. 1A). Each tub was filled with approximately 2 L of 12 ppt ASW and equipped with temperature and pH probes (Neptune Systems, San Jose, CA, USA), along with a submersible aquarium pump to circulate the tub water (Sensen, Zhoushan, Zhejian, China). The ambient tub was equipped with an airstone connected to an air pump (Tetra, The Woodlands, TX, USA) with a constant flow rate. The pH of the acidic tub was lowered by bubbling in CO_2_ via an airstone connected through a computer-controlled solenoid valve (Neptune Systems, San Jose, CA, USA) to a CO_2_ tank equipped with a regulator (Airgas, Radnor, PA, USA) set to ∼1 psi. The flow of CO_2_ into the acidic tub was controlled by an Apex aquarium controller system (Neptune Systems, San Jose, CA, USA), which tracked the pH of the water in the tub via the pH probe and adjusted the solenoid valve as needed to maintain a programmed pH of 7.40. Finally, both tubs were kept within a water bath that was maintained at 18°C by an aquarium chiller (Poafamx Amazon Store, Seattle, WA, USA).

Salinity, pH, and temperature were measured and recorded approximately daily to ensure the maintenance of experimental conditions. Seawater pH was measured and recorded using a handheld pH glass electrode (Mettler Toledo, Columbus, OH, USA), which was calibrated once a week using calibration solutions (pH 7 and 10) supplied by the probe manufacturer. Salinity and temperature were measured and recorded using a handheld meter (YSI Incorporated, Yellow Springs, OH, USA), and salinity was adjusted with deionized (DI) water as needed. Duplicate 50 mL seawater samples were collected from each tub every three days, then immediately sterilized with a 0.22 μm syringe filter (Sigma-Aldrich, Burlington, MA, USA) and stored at 4°C in conical tubes (Corning, Corning, NY, USA) until processing for total alkalinity (TA). TA was determined via titration using a Metrohm 905 Titrando (Metrohm, Herisau, Switzerland). Parameters of the carbonate system in the seawater samples [e.g. (carbonate), (bicarbonate), aragonite saturation state] were calculated from temperature, salinity, TA, and pH using the *seacarb* package in R (Gattuso et al., 2021).

### Experimental setup

Anemones were kept in 4 oz plastic treatment containers (Ziploc, San Diego, CA, USA) with 100 μm nylon mesh bottoms (Genesee Scientific Corporation, San Diego, CA, USA). Foam was attached around the rim of each container for floatation, and a total of six containers were placed in each tub. Female (*N*=28) and male (*N*=20) anemones were randomly distributed into three containers per sex per treatment, resulting in 3–4 males or 4–5 females per container (Fig. 1A). Anemones were fed approximately daily with fresh *Artemia nauplii* throughout the experiment.

### Anemone spawning

Anemones were spawned immediately prior to the initiation of the experiment to clear them of developing gametes (Marčeta et al., 2022; Rivera et al., 2021). Once the experiment was initiated, anemones were spawned approximately biweekly (weeks 2, 4, 7, 9, 11, and 13 of exposure) to correspond with the completion of consecutive gametogenic cycles (Rivera et al., 2021; Stefanik et al., 2013). Spawning was induced following the protocol developed by Stefanik et al. (2013) with minor variations. In short, anemones were removed from the treatment containers, placed in ambient (pH ∼7.72) 12 ppt seawater, and exposed to bright light at 24°C for approximately 12 h overnight. The anemones were then placed at room temperature (∼19–21°C), where they were monitored every 30 min for gamete release. For each spawning cycle, females were separated into individual plastic cups (∼25 ml) for the duration of the spawning protocol. Male anemones were either pooled by treatment (week 2), separated into groups of 3–4 in small glass bowls (week 4), or separated into individual plastic cups (weeks 7–13). The water in the tubs was replaced with 12 ppt ASW and equilibrated to the experimental conditions during each spawning session (15–18 h), after which anemones were returned to the corresponding treatment containers within the tubs.

### Female fecundity and egg size

Egg bundles were placed in individual wells of a 24-well plate with a plastic transfer pipette following spawning. Images of each bundle were collected using a Retiga R3 CCD camera (Meyer Instruments, Houston, TX, USA) attached to a Leica MZ12 dissecting microscope (Leica Camera, Wetzlar, Germany). The images were then analyzed for egg counts per bundle and egg sizes manually in Fiji (Schindelin et al., 2012). For egg sizes, a 1×1 mm grid was photographed as a size standard to calibrate images, the line tool was used to draw the diameter of each egg, and the length of the line was recorded. For weeks 2 and 4, 60 eggs were measured across all females, whereas 30 eggs per female were measured for weeks 7–13.

### Male fecundity

After the males spawned, the ASW containing live sperm (hereafter referred to as ‘sperm water’) was filtered through a 100 μm cell strainer (Corning, Corning, New York, USA) into a 50 ml conical tube to remove debris. Sperm concentrations were quantified using a hemocytometer (Marienfeld, Lauda-Königshofen, Germany) in weeks 2 and 4. Specifically, 1 ml of sperm water from each conical was transferred to a 1.5 ml tube and centrifuged once at 1500×***g*** for 5 min at 22°C; then, the supernatants removed, and the sperm pellets resuspended in 110 μl of 12 ppt ASW. Next, 10 μl aliquots of the concentrated sperm from each treatment were loaded separately onto a hemocytometer, and cells were counted under 10× magnification according to the manufacturer's instructions. Sperm concentrations were divided by the number of males in each container to determine the average number of sperm produced per male. For weeks 7–13, sperm concentration was measured for each individual male anemone with a Guava^Ⓡ^ easyCyte™ HT flow cytometer (MilleporeSigma, St. Louis, MO, USA) in triplicate (as technical replicates) in accordance with the manufacturer's instructions.

### Sperm performance

MMP was measured using the fluorescent dye JC-1 (Thermo Fisher Scientific, Waltham, MA, USA) in weeks 11 and 13. Sperm were pooled by treatment and 1 ml subsamples were incubated with 20 μM JC-1 for 15 min in the dark. A separate aliquot was treated with carbonyl cyanide m-chlorophenyl hydrazone (CCCP; Cell Signaling Technology, Danvers, MA, USA) at a final concentration of 50 μM for 15 min followed by JC-1 as a negative control. Sperm were then centrifuged at 1500×***g*** for 5 min to remove excess dye, resuspended 12 ppt ASW at a concentration of 5×10^5^ sperm ml^−1^, and distributed in triplicate into a 96-well plate. The plate was kept dark and loaded into a Guava^Ⓡ^ easyCyte™ HT flow cytometer (MilleporeSigma, St. Louis, MO, USA). Samples were excited at 488 nm, and fluorescence was detected at two wavelengths: GRN-B (525/30 nm) and YEL-B (583/26 nm). Each well was read for at least 60 s, resulting in more than 1.5×10^4^ cells quantified per well. Using in Guava^Ⓡ^ InCyte, plots of green versus yellow fluorescence produced by samples treated with both CCCP and JC-1 were used for gating as in Henley et al. (2021), then gates were used on all other sample plots to quantify percentages of sperm with high MMP (Fig. S1).

Fertilization rates were quantified in weeks 2, 4, and 9. Sperm water was combined into a single pool per treatment and each pool was diluted to the same concentration, which differed between spawning dates (Table S2). Each pool was then serially diluted 1:2 in 12 ppt ASW to obtain four different sperm concentrations. Egg bundles were separated into six-well plates, with 1–2 bundles per well, and 6 mL of sperm water from the corresponding experimental treatment was pipetted into each well, with three replicates per sperm concentration per treatment for a total of 12 wells per pH treatment. At 3 h post-fertilization (HPF), each well was photographed using a dissecting microscope with a camera attachment, and the number of fertilized and unfertilized eggs were counted manually. Fertilized eggs were identified by their conspicuous bumpy appearance, which is a result of initial cell divisions, whereas unfertilized eggs maintained a round shape. For each well, the percentage of fertilized eggs was calculated by dividing the number of fertilized eggs by the total number of eggs in the well.

### Larval performance

Following fertilization assays, the resulting embryos were held at room temperature (∼19–21°C) to allow them to develop into swimming (planula) larvae. Water changes were performed at 24 HPF by aspirating water from each well (12 per treatment) followed by addition of newly made 12 ppt ASW. At 3 days post-fertilization (DPF), wells were examined under a dissecting microscope and the number of swimming larvae were counted along with the number of surviving non-motile larvae; unfertilized eggs and dead larvae had begun to visibly disintegrate and were clearly distinguishable from live but non-motile larvae. The percentage of larvae in the planula stage at 3 DPF was calculated by dividing the number of swimming larvae by the total number of surviving larvae. At 7 DPF, wells were again examined, and the number of larvae that had undergone settlement and metamorphosis were counted, along with the total number of surviving larvae. The percentage of larvae settled was calculated by dividing the number of settled larvae by the number of surviving larvae (planulae and settled) for each well.

To quantify larval respiration rates, sperm and eggs were first combined by treatment in glass finger bowls in weeks 11 and 13. At 3 DPF, swimming larvae from each parental treatment were pipetted in three groups of ten (week 11) or nine groups of 15 (week 13) into wells of a 24-well plate equipped with oxygen sensor spots (Loligo Systems, Viborg, Denmark). Wells containing larvae as well as larvae-free wells containing water from the fertilization bowl as a bacterial control (‘blanks’; *N*=3 in week 11; *N*=6 in week 13) were filled to capacity (80 μL) with 12 ppt ASW and sealed with an adhesive plate cover before being placed on a PreSens SensorDish^Ⓡ^ Reader (Precision Sensing, Regensburg, Germany), which was previously calibrated according to the manufacturer's instructions. Dissolved oxygen concentrations in each well were read every 15 s for at least 1 h, during which no wells experienced near or total oxygen depletion. The rate of oxygen consumption over time was determined from the slopes of linear regressions of oxygen levels multiplied by the volume of the wells. The average oxygen consumption rate for the blank wells was subtracted from the larval rates, which were then converted to nmol O_2_ minute^−1^ larva^−1^.

Larval heat tolerance was quantified at 3 DPF using a protocol modified from Rivera et al. (2021). Larvae from each parental treatment were individually pipetted into polymerase chain reaction (PCR) strip tubes (*N*=32 larvae treatment^−1^ temperature^−1^). Larvae were then exposed to one of a range of peak temperatures between 39–43°C in 0.5-degree increments. MiniAmp thermal cyclers (Thermo Fisher Scientific, Waltham, MA, USA) were used for heat ramps, which were programmed as follows: (1) 1 min at 25°C; (2) 4 min at 30°C; (3) 4 min at 38°C; (4) 1 h at the peak temperature (39–43°C); (5) 4 min at 38°C; (6) 4 min at 30°C; (7) infinite hold at 22°C. Strip tubes containing larvae were capped, randomly assigned to positions in the thermal cyclers for the heat ramp, and then removed and uncapped as soon as the cool-down ramp was complete. Following uncapping, tubes were placed in the dark at 18°C for 48 h, then larvae were transferred with a multichannel pipette to a 96-well plate and examined for survival (no clear disintegration of tissue) under a dissecting microscope. The percentage of larvae surviving after exposure to each peak temperature was calculated as the number of larvae surviving divided by the total number of larvae exposed to each temperature.

### Adult respiration

Respiration rates of adult anemones (*N*=9 ambient males, 13 ambient females, eight acidic males, 12 acidic females) were measured 5 days after the week 13 spawning. Anemones were transferred individually to 4 mL glass vials equipped with oxygen sensor spots (Loligo Systems, Viborg, Denmark), which were filled to capacity with the respective treatment water and sealed. Several vials were also filled with only ASW to serve as a bacterial control. Vials were placed on a calibrated PreSens SDR as described above, and oxygen concentrations were recorded every 15 s for at least 1 h. Oxygen consumption rates were calculated for each individual as described above and converted to nmol O_2_ minute^−1^ anemone^−1^.

### Statistical analyses

All statistical analyses and figure generation were performed using R 4.0.5 (R Core Team, 2022) in RStudio. For data pertaining to the response variables carbonate chemistry, male fecundity, sperm MMP, female fecundity, egg size, larval planulation, larval settlement, and larval respiration, data were first analyzed using linear models with treatment, date, and their interaction as independent variables. For larval planulation and settlement, both raw and arcsine-square root transformed data were compared to ensure no difference in statistically significant model terms. For fertilization data, sperm concentration was also included as an independent variable; a separate linear model was created relating the number of eggs within a given egg bundle to the average size of eggs in the same bundle. For larval heat tolerance, generalized linear models (GLMs) were created from binary survival data for each treatment, and related survival to temperature. A combined GLM was also created for the data to assess the significance of treatment and the interaction between treatment and temperature. All models with interactive terms were first analyzed for significance with ANOVAs using Type III sums of squares; interaction coefficients were dropped from the models when they lacked significance (*P*>0.05) followed by confirmation of a superior model fit using Akaike information criterion corrected for small sample sizes (AICc). For any revised models lacking interaction terms, significance was reanalyzed using Type II ANOVAs. For models with significant *P*-values (*P*<0.05) for any term, effects were further interrogated with Tukey's HSD post-hoc test. A final summary of all models, post-hoc tests, and relevant significance information can be found in Table S1. The following packages were used: *ggplot2* (Wickham, 2016), *ggpubr* (Kassambara, 2020), *plotrix* (Lemon, 2006), *tidyverse* (Wickham et al., 2019), *tidyr* (Wickham and Girlich, 2022), *dplyr* (Wickham et al., 2022), *car* (Fox and Weisberg, 2019), *emmeans* (Lenth et al., 2022), *Rmisc* (Hope, 2022), *oce* (Kelly et al., 2022), *lubridate* (Spinu et al., 2021), *mgcv* (Wood, 2022), and *MuMIn* (Bartoń, 2022).

## Supplementary Material

10.1242/biolopen.059746_sup1 Supplementary information
