# Supplementary material for: Parental exposure to ocean acidification impacts gamete production and physiology but not offspring performance in Nematostella vectensis
Source: Biol Open. 2023 Feb 28;12(3):bio059746. doi: 10.1242/bio.059746 (PMC10003076; doi:10.1242/bio.059746)
Supplement: Supplementary information [file biolopen-12-059746-s1.pdf]

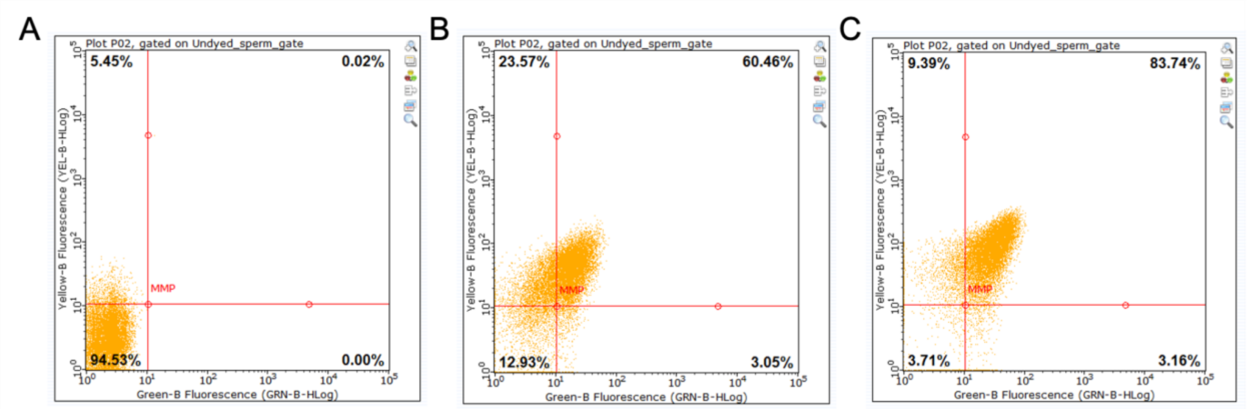

Fig. S1. Example plots for flow cytometry analysis of sperm MMP. A) Representative plot of JC-1 yellow versus green fluorescence for sperm treated with the mitochondrial inhibitor CCCP followed by JC-1. Red lines depict the gate that was drawn based on multiple CCCP samples and applied to other plots. B) Representative plot of JC-1 yellow versus green fluorescence for sperm treated with only JC-1, also depicting the JC-1 gate and percentages of sperm falling in gate quadrants. C) Additional representative plot of JC-1 yellow versus green fluorescence for sperm treated with only JC-1, which shows a higher percentage of sperm with high MMP (top right quadrant) compared to panel B.

Table S1. Summary of statistical analyses.

DVar	Model	Test	IVar	df	F value	p-value	Tukey	Tukey p-value
pH	lm(pH ~ Tr)	Type II ANOVA	Tr	1	354.32	< 0.001	Am > Ac	< 0.001
pCO_2	lm(pCO2 ~ Tr)	Type II ANOVA	Tr	1	39.621	< 0.001	Am < Ac	< 0.001
TA	lm(TA ~ Tr)	Type II ANOVA	Tr	1	4.867	0.032	Am < Ac	0.032
Ωarag	lm(Ωarag ~ Tr)	Type II ANOVA	Tr	1	30.146	< 0.001	Am > Ac	< 0.001
Female fecundity	lm(FF ~ Tr*D)	Type III ANOVA	Tr	1	0.278	0.599		
			D	5	28.136	< 0.001		
			Tr*D	5	2.450	0.036	W2Am > W2Ac	< 0.001
Egg size	lm(ES ~ Tr*D	Type III ANOVA	Tr	1	0.248	0.619		
			D	5	26.411	< 0.001		
			Tr*D	5	9.834	< 0.001	W4Am < W4Ac	0.002

Biology Open • Supplementary information

							W9Am < W9Ac	< 0.001
							W11Am < W11Ac	< 0.001
FF vs. size	lm(FF ~ ES)	Type II ANOVA	ES	1	13.029	< 0.001 (R = -0.34)		
Male fecundity	lm(MF ~ Tr + D)	Type II ANOVA	Tr	1	0.072	0.789		
			D	5	1.581	0.182		
Sperm MMP	lm(MMP ~ Tr*D)	Type III ANOVA	Tr	1	260.635	< 0.001		
			D	1	122.743	< 0.001		
			Tr*D	1	66.585	< 0.001	W11Am < W11Ac	< 0.001
							W13Am < W13Ac	0.002
Fert	lm(fert ~ Tr + conc)	Type II ANOVA	Tr	1	4.535	0.037	Am < Ac	0.037
			Conc	1	5.890	0.018		
Larval Planula	lm(PP ~ Tr + D)	Type II ANOVA	Tr	1	2.129	0.152		

Biology Open • Supplementary information

			D	1	94.640	< 0.001
Larval Resp.	lm(LR ~ Tr + D)	Type II ANOVA	Tr	1	0.080	0.781
			D	1	0.004	0.954
Larval HT	glm(Surv ~ Tr + T)	Type II ANOVA	Tr	1	2.62 (ChiSq)	0.106
			T	1	510.69 (ChiSq)	< 0.001 (R = -0.79)
Larval Settle.	lm(PS ~ Tr + D)	Type II ANOVA	Tr	1	1.015	0.319
			D	1	48.213	< 0.001
Adult Resp.	lm(AR ~ Tr + Sex)	Type II ANOVA	Tr	1	4.493	0.051
			Sex	1	1.654	0.218

DVar = dependent variable; IVar = independent variable; Tr = treatment; Am = ambient; Ac = acidic; TA = total alkalinity; Ωarag = aragonite saturation state; FF = female fecundity; D = date; W = week; ES = egg size; MF = male fecundity; fert = fertilization; conc = sperm concentration; PP = percent of larvae in planula stage; LR = larval respiration rate; HT = heat tolerance; surv = survival; T = temperature; PS = percent of larvae settled; AR = adult respiration rate.

Table S2. Starting sperm concentrations for fertilization assays.

Week	Sperm Concentration (sperm mL^{-1})
Week 2	128,750
Week 4	1,250,000
Week 7	489,516
Week 9	1,600,500
